# Supplementary material for: The Systems Biology Research Tool: evolvable open-source software
Source: BMC Syst Biol. 2008 Jun 29;2:55. doi: 10.1186/1752-0509-2-55 (PMC2446383; doi:10.1186/1752-0509-2-55)
Supplement: Additional file 1 — SBRT Archive. An archive of the current version of the Systems Biology Research Tool. [file 1752-0509-2-55-S1.zip › sbrt-1.4.0/doc/developers_guide/api/sbrt/kernel/fba/class-use/IrrevRxnFluxomeV1.html]

Uses of Class sbrt.kernel.fba.IrrevRxnFluxomeV1


|  |  |  |
| --- | --- | --- |
| **Overview** \| **Package** \| **Class** \| **Use** \| **Tree** \| **Deprecated** \| **Index** \| **Help** | |  |
| PREV   NEXT | **FRAMES**    **NO FRAMES**     **All Classes** |  |


---


## **Uses of Class sbrt.kernel.fba.IrrevRxnFluxomeV1**

| Packages that use IrrevRxnFluxomeV1 | |
| --- | --- |
| **sbrt.kernel.fba** | Provides classes and interfaces for creating objects relevant to Flux Balance Analysis. |

Uses of IrrevRxnFluxomeV1 in sbrt.kernel.fba

| Methods in sbrt.kernel.fba that return IrrevRxnFluxomeV1 | |
| --- | --- |
| `static IrrevRxnFluxomeV1` | `IrrevRxnFluxomeV1.make(java.util.Collection<java.lang.String> reactionNames, IrrevRxnFluxome fluxome)`             Returns an irreversible reaction fluxome composed of the reactions in the provided fluxome with the names in the provided collection. |
| `IrrevRxnFluxomeV1` | `IrrevRxnFluxomeV1.replaceConstraints(FluxConstraints fluxConstraints, double constraintTolerance)`             Returns a copy of this fluxome, but with all constraints replaced by those provided. |

---


|  |  |  |
| --- | --- | --- |
| **Overview** \| **Package** \| **Class** \| **Use** \| **Tree** \| **Deprecated** \| **Index** \| **Help** | |  |
| PREV   NEXT | **FRAMES**    **NO FRAMES**     **All Classes** |  |


---
